# Patterns of Coevolutionary Adaptations across Time and Space in Mouse Gammaretroviruses and Three Restrictive Host Factors

**DOI:** 10.3390/v13091864

**Published:** 2021-09-18

**Authors:** Guney Boso, Oscar Lam, Devinka Bamunusinghe, Andrew J. Oler, Kurt Wollenberg, Qingping Liu, Esther Shaffer, Christine A. Kozak

**Affiliations:** 1 Laboratory of Molecular Microbiology, National Institute of Allergy and Infectious Diseases, Bethesda, MD 20892, USA; guney.boso@nih.gov (G.B.); Oscar.lam@nih.gov (O.L.); devinka123@gmail.com (D.B.); liuqing@niaid.nih.gov (Q.L.); esther.shaffer@nih.gov (E.S.); 2 Bioinformatics and Computational Biosciences Branch, Office of Cyber Infrastructure and Computational Biology, National Institute of Allergy and Infectious Diseases, Bethesda, MD 20892, USA; andrew.oler@nih.gov (A.J.O.); wollenbergk@niaid.nih.gov (K.W.)

**Keywords:** mouse gammaretroviruses, restriction factors, endogenous retroviruses, positive selection, coevolution, *Fv1* restriction, XPR1 virus receptor, CAT1 virus receptor, geographic mosaics

## Abstract

The classical laboratory mouse strains are genetic mosaics of three *Mus musculus* subspecies that occupy distinct regions of Eurasia. These strains and subspecies carry infectious and endogenous mouse leukemia viruses (MLVs) that can be pathogenic and mutagenic. MLVs evolved in concert with restrictive host factors with some under positive selection, including the XPR1 receptor for xenotropic/polytropic MLVs (X/P-MLVs) and the post-entry restriction factor *Fv1*. Since positive selection marks host-pathogen genetic conflicts, we examined MLVs for counter-adaptations at sites that interact with XPR1, *Fv1*, and the CAT1 receptor for ecotropic MLVs (E-MLVs). Results describe different co-adaptive evolutionary paths within the ranges occupied by these virus-infected subspecies. The interface of CAT1, and the otherwise variable E-MLV envelopes, is highly conserved; antiviral protection is afforded by the *Fv4* restriction factor. XPR1 and X/P-MLVs variants show coordinate geographic distributions, with receptor critical sites in envelope, under positive selection but with little variation in envelope and XPR1 in mice carrying P-ERVs. The major *Fv1* target in the viral capsid is under positive selection, and the distribution of *Fv1* alleles is subspecies-correlated. These data document adaptive, spatial and temporal, co-evolutionary trajectories at the critical interfaces of MLVs and the host factors that restrict their replication.

## 1. Introduction

The classical inbred strains of laboratory mice carry mouse leukemia viruses (MLVs) of three host range groups: ecotropic, xenotropic, and polytropic (E-MLVs, X-MLVs, P-MLVs) [1,2,3]. These gammaretroviruses are found either as infectious retroviruses (XRVs) or as endogenous retroviruses (ERVs), which are viral DNA copies inserted into host chromosomes during past infections. These inbred strains are intersubspecific hybrids of three house mouse subspecies, *Mus musculus musculus*, *M. m. castaneus*, and *M. m. domesticus* [4]. All of these subspecies carry MLVs; polytropic ERVs (P-ERVs) are predominate in Western European and North African *M. m. domesticus*, while xenotropic and ecotropic XRVs and ERVs (X- and E-MLVs) are found in Eurasian *M. m. castaneus* and *M. m. musculus* [5]. These subspecies, and the viruses they carry, likely originated in the Indian subcontinent and the neighboring Iranian plateau [6] but followed humans engaged in migration or trade and now have a global distribution with largely nonoverlapping geographic ranges in Eurasia that can be separated by defined hybrid zones [7].

The various laboratory strains and wild mouse subspecies differ in their susceptibility to MLVs, and to virus-induced disease, due to host factors that can inhibit virus replication at different stages of the virus life cycle, including entry, reverse transcription, transport to the nucleus, transcription, and budding [8]. Some of these factors have no known function other than virus restriction, while others serve important host functions that also facilitate virus replication but can have restrictive polymorphic variants. These factors act to mitigate the consequences of exposure to infectious and endogenous MLVs that can be pathogenic and mutagenic. Polymorphisms in these host restriction factors can alter virus restriction patterns, and viruses can acquire adaptive mutations at sites that interact with these antiviral host factors. This evolutionary “arms race”, described by the Red Queen hypothesis [9], notes that both the host and pathogen have to change continuously to keep up with the newly acquired antagonistic adaptations in their adversaries. This process has produced adaptive changes in the post-entry restriction factor *Fv1* and in the XPR1 receptor for X/P-MLVs [10,11]. Both genes are under positive selection, in rodents, that is centered on sites critical for virus restriction. These analyses, however, present a limited picture of this host-pathogen co-evolutionary history as they have largely focused on the host partner in these conflicts.

The divergence of *M. musculus*, into subspecies that are geographically separated, also provides a unique opportunity to describe any phylogeographic evolutionary patterns in these viruses and host restriction factors. Antagonistic host-pathogen interactions can drive evolutionary changes on spatial, as well as temporal, scales, generating genetic diversity in physically separated populations. Coevolution can thus proceed along different trajectories in separated populations, creating distinctive geographic mosaics as described by the Geographic Mosaic Theory of Evolution [12].

Here, we focused our attention on the divergence of MLVs and ERVs in *M. musculus,* examining the capsid target of *Fv1* and the receptor binding domains (RBDs) of X/P- and E-MLV *envs*, which utilize the XPR1 and CAT1 receptors, respectively. We looked for positive or purifying selection at these protein interfaces and for any previously overlooked restriction factor variants. We describe the geographic distribution of MLV *env* variants and *Fv1* alleles in natural house mouse populations. We show evidence of reciprocal positive selection at the MLV sites that interact with *Fv1* and the XPR1 receptor, but we find a “cold spot” in European *M. m. domesticus* where there is no obvious sign of conflict between the MLVs they harbor and XPR1. We also report that, despite extensive sequence variation in the E-MLV RBD, these MLVs show no adaptive changes in the receptor binding pocket nor is there variation in the receptor critical region of the *M. musculus* CAT1 suggesting that other factors, such as the *Fv4* restriction gene in some virus-infected populations, help mitigate the consequences of infection.

## 2. Materials and Methods

### 2.1. Sources of Mouse DNAs and RNAs

Sources of mice and DNAs are listed in Table S1. Some DNAs were isolated from mice maintained in our laboratory or obtained from M. Potter (NCI, Bethesda, MD, USA) and from S. Rasheed (University of Southern California, Los Angeles, CA, USA). Additional DNAs from wild-caught or wild-derived mice were purchased from The Jackson Laboratory (Bar Harbor, ME, USA) and from RIKEN BioResource Center (Tsukuba, Japan) with assistance from Drs. Toshihiko Shiroishi and Takehide Murata, or were obtained from R. Abe (Naval Medical Research Institute, Bethesda, MD, USA) and from S. Chattopadhyay and H. Morse III (NIAID, Bethesda, MD, USA).

### 2.2. Primers, PCR, Cloning and Sequencing

Primers for PCR (Table S2) were designed to amplify the full-length or C-terminal half of *Fv1*, MLV subtype specific segments, and the full length CAT1 receptor or genomic CAT1 segments, including exons 3-5, which cover the receptor determining region. Selected PCR products were cloned into pCR2.1-TOPO (Thermo Fisher, Waltham, MA, USA) and sequenced (Datafile S1). Other sequences used for analysis included previously reported mouse ERVs and XRVs and genes for CAT1 and *Fv1* from various *Mus musculus* subspecies and other rodents (Table S3) [13,14,15].

### 2.3. Identification of Variants in Fv1 and Slc7a1(CAT1) Genes in M. m. castaneus

Aligned sequence reads (BAM formatwere obtained for ten wild *M. m. castaneus* animals trapped in different locations in northwest India from Daniel Halligan and P. Keightley (University of Edinburgh) [16]. Reads were subset for the *Fv1* and *CAT1* genes using SAMtools (version 0.1.18) [17], coordinates chr4:147242588-147244967 or chr5:149138986-149211480 according to the mm9/NCBIM37 reference assembly. For *Fv1*, files for each strain were converted to FASTQ format with bam2fastx (from TopHat package [18]) and aligned to the reference using BWA MEM [19] (version 0.7.5a-r405) For both genes, duplicates were marked with MarkDuplicates (http://broadinstitute.github.io/, accessed on 22 December 2014; version 1.75), and variants were called using GATK (version 3.3) Best Practice methods [20,21,22], including indel realignment, single-sample calling with HaplotypeCaller and joint genotyping with GenotypeGVCFs. To further refine the indels at the C-terminus of *Fv1*, reads were assembled with SOAPdenovo2 [23] (version LINUX-generic-r240) with kmer setting ranging from 25 to 61 and resulting contigs with coverage > 1 were assembled using Lasergene SeqMan (DNASTAR, Inc, Madison, WI, USA). Variant effects were annotated using VEP [24] (archived tool at http://may2012.archive.ensembl.org/tools.html, accessed on 22 December 2014). Linkage/phasing of variants within a strain was determined by HaplotypeCaller or manual inspection of paired reads using IGV [25], SAMtools, and BLAT [26] at the UCSC Genome Browser (http://genome.ucsc.edu/, accessed on 22 December 2014) [27].

### 2.4. Identification of the Subspecies Origin of Fv1 

We used the Mouse Phylogeny Viewer (MPV) at the University of North Carolina (http://msub.csbio.unc.edu, accessed on 14 March 2021) [28] to identify the subspecies of origin of *Fv1* alleles using chromosome coordinates from the NCBI37/mm9 reference assembly. This browser uses a set of diagnostic single-nucleotide polymorphisms (SNPs) to define the local subspecific origin along each autosome and the X chromosome for a set of 100 classical laboratory strains and 98 wild-derived and wild-caught mice. MPV also identifies regions of haplotype identity for the inbred strains and the SNP variants that define those regions.

### 2.5. Phylogenetic and Positive Selection Analyses

The sequence of the *Slc7a1* (CAT1) gene and segments of *env* and *gag* from E- and X/P-MLVs were aligned using MUSCLE as implemented in Geneious 10.0.9 using default settings [29,30]. *Env* genes were analyzed using a set of full-length genes as well as a larger set covering the RBD*env* that includes newly sequenced wild mouse ERVs and previously published sequences [31]; the capsid analysis emphasized E-XRVs tested for *Fv1* sensitivity. Phylogenetic trees were generated using the RaxML program with the GTR+G+I model and 500 bootstraps for branch support [32].

For maximum-likelihood analysis of codon evolution, we used codeml of PAML 4.9, in addition to four programs on the DataMonkey Web server: MEME, FUBAR, SLAC, and FEL [33,34]. Alignments for RBD*env* of E-MLVs and the CAT1 genomic segments were trimmed to the shortest sequence and were manually inspected to exclude indels found in more than a few species, as recommended by the developers of PAML. Primer sequences used for amplification were excluded from the analysis. To calculate branch-specific *dN/dS* values, we utilized the free-ratio model in codeml of PAML, and to identify specific codons under positive selection, the F61 and F3x4 codon frequency models were used with different initial seed values of ω. Likelihood ratio tests were performed to compare two pairs of site-specific models: M1, a neutral model that does not allow positive selection, with M2, a model that allows positive selection; M7, another neutral model with beta distribution of *dN*/*dS* values, with M8, a positive-selection model with beta distribution. In each case, chi-square analysis was done, and a model that allowed positive selection was a significantly better fit to the data than the null (neutral) model (*p* < 0.05). Posterior probabilities of codons under positive selection were inferred using the BEB algorithm in the M8 model [35]. Alternative tests for positive-selection analyses used the MEME, FEL, SLAC, and FUBAR programs with recommended settings [36] and the positively selected residues with *p* < 0.1 were chosen.

## 3. Results and Discussion

### 3.1. E-MLVs and Their CAT1 Receptor

#### 3.1.1. E-MLVs

**Figure 1:**
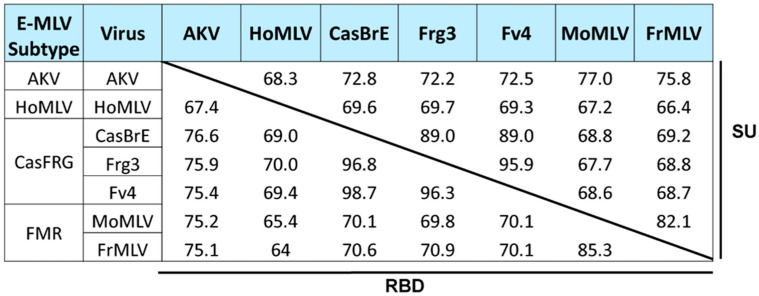
Variation in E-MLV *env* genes. The matrix shows percent sequence identity of representative E-MLVs in SU and RBD.

E-MLVs infect cells of the mouse and some related rodents. Decades of studies identified three E-MLV *env* subtypes carried by laboratory and/or wild mice (AKV, Cas/Frg, HoMLV), and a fourth group consisting of the laboratory-derived FMR strains (Friend, Moloney, and Rauscher). E-MLV genomes appear to be recombinants with different E-MLV *env* genes embedded in non-ecotropic *gag-pol* backbones [15]. Despite their shared ecotropism, the surface (SU) domains of the *env* subtypes are only 66.4–77% identical, with similar identities in the RBD—the first 236 codons of the SU (Figure 1). While many of these subtype differences localize to the proline-rich region [15], most polymorphisms within RBD are concentrated in the VRA and VRB variable domains [37].

**Figure 2:**
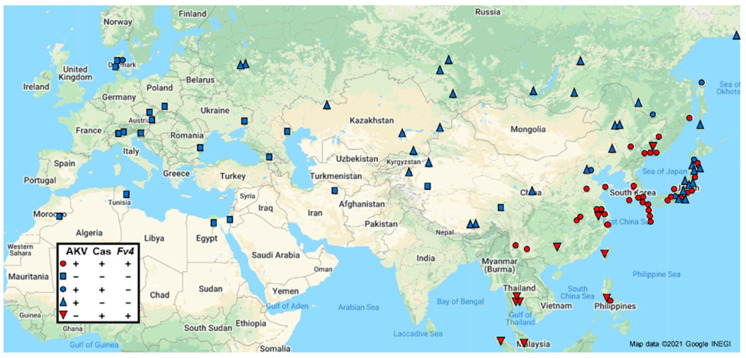
Distribution of AKV and Cas/Frg E-MLV *env* subtypes and the *Fv4* restriction gene *env* in Eurasian *M. musculus*.

Here we typed a large panel of wild-caught and wild-derived mice (Table S1) by PCR to identify the presence and geographic distribution of AKV, Cas/Frg, and HoMLV ERVs. Primers were specific for the *gag-pol* and *env* regions of HoMLV, the *env* genes of AKV and Cas/Frg, and the virus-cell junction and empty insertion site of the Cas/Frg *env*, integrated at *Fv4,* a restriction factor that blocks E-MLV replication [38] (Table S2). Consistent with limited earlier analyses based on Southern blotting [5,39,40], E-ERV *envs* were restricted to some *M. musculus* subspecies indicating their recent acquisition (Figure 2). No mice carry HoMLV except the original source, the Eastern European mouse *M. spicilegus,* in which HoMLV did not endogenize [41], while the AKV, Cas/Frg, and *Fv4 envs* show broad, but distinctive, geographic distributions, although none are found in *M. m. domesticus* of Western Europe and North Africa (Figure 2). AKV E-ERVs, carried by many classical inbred strains [1,42], are found in *M. m. musculus* populations in southern China, Russia, and Korea, as well as Japan, where house mice are natural hybrids of *M. m. musculus* and *M. m. castaneus* and are often designated as a separate subspecies, *M. m. molossinus* [43]. Cas/FrgMLVs are found in Korea and SE China as well as the various countries of SE Asia (Figure 2).

The Cas/Frg-derived protective factor *Fv4* is an expressed *env* gene carried by all mice from SE Asia, Eastern China, and Korea (Figure 2), indicating that this ERV was domesticated in *M. m. castaneus*. We also found this highly advantageous antiviral gene in about half of the mice sampled in Japan and in all seven of the mice trapped in Lake Casitas, CA. *Fv4* is thus present where mice of two different subspecies were artificially introduced through commensalism: *M. m. castaneus* and *M. m. musculus* in Japan, and *M. m. castaneus* and *M. m. domesticus* in California [5,44]. Although effective against AKV MLVs in laboratory mice [45], *Fv4* has not successfully crossed the hybrid zone separating *M. m. castaneus* and *M. m. musculus* in China, a barrier largely defined by the Yantgze River [46]. This suggests the possibility that AKV type E-MLVs are not a major survival threat in *M. m. musculus*, likely due to the fact that lymphomagenesis by E-MLVs requires recombination with P-ERVs [47], which are not carried by *M. m. musculus* [5]. The Cas/Frg E-MLVs present in California and *M. m. castaneus* can also induce neurological diseases without alteration by recombination [48], but they are subject to *Fv4* restriction.

**Figure 3:**
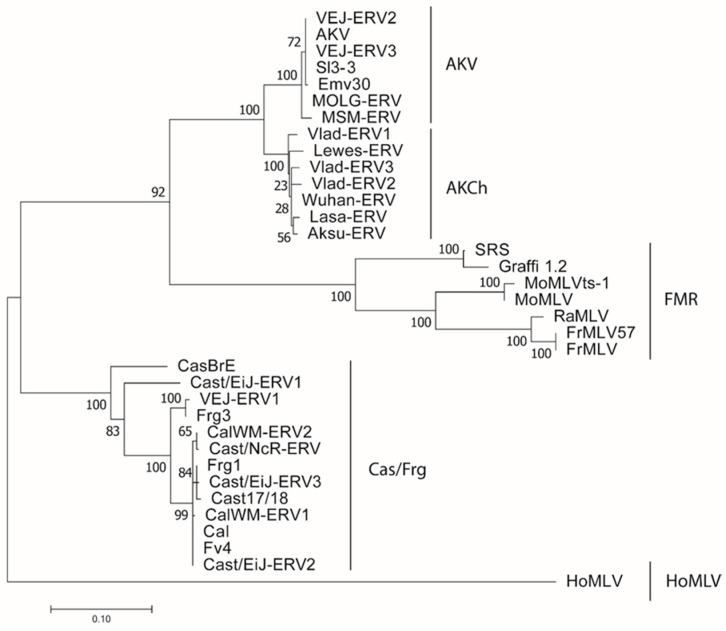
Phylogeny of E-MLV *env* RBD sequences. Sequences corresponding to the RBD of the indicated E-MLVs were aligned and a maximum likelihood tree was generated using RaxML with 500 replicates. Bootstrap values are shown at each node. The tree was midrooted. Lines on the right side of the tree identify the five E-MLV subtypes.

We sequenced *env* genes from various wild mice to screen for additional E-*env* variants. Sequence alignments and a phylogenetic tree of the *env* or RBD sequences show five clades with distinctive patterns of shared substitutions (Figure 3 and Figure S1); these clades correspond to the three known wild mouse subtypes and the FMR viruses, and they identify a novel AKV-related mouse subtype, AKCh E-MLV, found in mice trapped in regions occupied by *M. m. castaneus* in Wuhan, in the S. Central city of Lasa, near the western Chinese border and in Russia near the eastern Chinese border. In RBD, AKCh MLVs are 99% identical but 90% identical to AKV MLVs. The Cas/Frg *env* genes were found in *M. m. castaneus* and in mice trapped in S. California, and AKV MLV *env*s were identified in *M. m. molossinus*.

The N-terminal end of the MLV RBD containing VRA has been linked to receptor choice [37]. Virus entry into susceptible mouse cells is governed by seven RBD residues [49], three of which (S84, D86, W102) form a binding pocket for the CAT1 receptor [50]. Substitutions at six of these seven sites in FMR isolates can alter infectivity and/or induce cytopathic syncytia in some susceptible cells (Table 1).

Despite the extensive sequence variation in the E-MLV *env* genes, two of the three binding pocket residues, D86 and W102, are invariant in all naturally occurring *env* genes, while the third site shows the conservative substitution S84A in Cas/Frg E-MLVs; this substitution is also found in the FrMLV variant F-S MLV, where it modifies, but does not compromise, receptor use [51] (Table 1). The other sites that can influence virus entry show a few substitutions found in XRVs or that are lineage specific. S76 and/or S77 are deleted in MoMLV and HoMLV, and substitutions are found in the Cas E-MLVs (S76D, S77K). The only wild mouse virus substitution at E116 (E116G) is found in HoMLV, which is replication competent.

**Figure 4:**
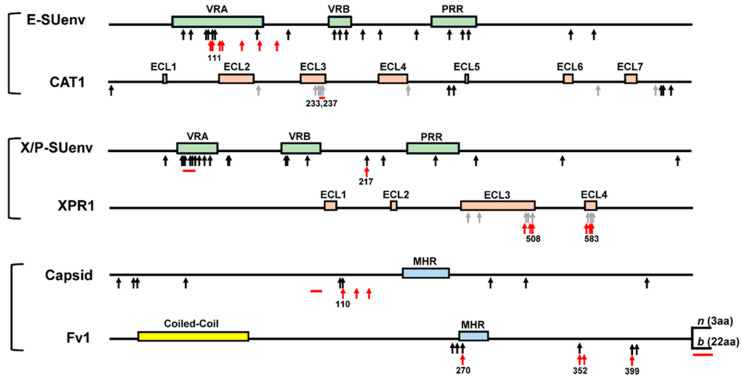
Positive selection and critical functional sites in retroviral restriction factors and their virus gene targets. Brackets define interacting virus/host pairs. Sites of positive selection in MLVs, and in host genes, are identified by black arrows; sites identified in rodent orthologs by gray arrows; red arrows and bars mark residues or regions critical for restriction or that are restriction targets. Numbers identify residues involved in virus/host interactions that are also under positive selection. VRA, VRB, variable domains; PRR, proline rich region; ECL, extracellular loop; MHR, major homology region; *n* and *b* identify alternative C-termini of *Fv1*.

Two sets of *env* sequences, 11 full length and 28 segments of the RBD*env*, were separately tested for evidence of diversifying/positive or purifying/negative selection, based on the ratio of the rate of nonsynonymous (*dN*) versus synonymous (*dS*) changes. Laboratory derived FMR strains were excluded from this analysis since their evolutionary path was likely different than the other groups. Using the maximum likelihood models in the codeml program of PAML4, and the MEME, FEL, FUBAR, and SLAC programs in the datamonkey webserver, we identified an excess of nonsynonymous mutations (*dN*/*dS* > 1, *p* < 0.1) at 26 sites scattered throughout the sequence, 15 of which were identified in the full *env* analysis, and 11 additional sites were found in the analysis based on RBD (Figure 4, Table S4). There are 14 of the 26 sites in the RBD, which includes VRA and VRB, with seven in VRA (Figure 4, Table S4), four of which form a patch surrounding the deletion, which alters MoMLV entry (Table 1), but none are at the residues that form the binding pocket. The various replacement mutations at all sites under positive selection are found in infectious E-MLVs.

These data show that the E-MLVs all use the CAT1 receptor but the extensive diversity among their *env* genes does not impact sites involved in virus entry. While it is possible the different *env* subtypes are independent acquisitions, it is more likely they derive from cumulative adaptations to their different mouse hosts, resulting in the emergence of three distinctive E-MLV *envs* in Eurasia and a fourth set of laboratory-derived FMR viruses. The limited historical record shows that FMR viruses were isolated from passaged tumors that probably arose in fancy mice carrying AKV E-MLVs; this virus was likely present in the fancy mouse progenitors of laboratory strains, which included the Japanese waltzer mouse [57]. These tumors had undergone forced passage through many mouse hosts for decades before virus isolation, the first report of which was by Gross in 1951 [58] who passed such tumors and tumor filtrates in mice as well as rats; this work inspired other investigators to attempt virus isolation from other transplantable tumors. MoMLV, for example, was isolated by John Moloney in 1960 [59] from Sarcoma 37, a tumor that had been passaged in mice since 1907 [60], before the development of inbred strains. Similarly, FrMLV was isolated by Charlotte Friend, in 1957, from an NIH Swiss mouse inoculated with Ehrlich ascites cells [61]. The *env* sequence variants in these FMR viruses were thus acquired over a short time frame. Similarly, in natural populations, the acquired changes in the Eurasian and FMR Env proteins differ from each other and from the Eastern European HoMLV. The evolutionary pressures responsible for the observed *env* variation are, therefore, unrelated to receptor interactions but may result from evasive changes in response to host immune defenses [62]. Env glycoproteins stud the outside of the virion and are thus vulnerable to host defenses, and the majority of sites under positive selection are in RBD, the most prominently exposed domain of the viral Env.

#### 3.1.2. CAT1 Receptor

All E-XRVs use the CAT1 receptor, encoded by *Slc7a1*, and the active receptor sites have previously been localized to a patch of critical residues (_232_NVKYGE_237_) in the third of its seven extra cellular loops (ECL3) [63] (Figure 4). CAT1 is an amino acid transporter that functions as an E-XRV receptor only in *Mus* species and some other rodents, including rats and hamsters, where virus entry can be restricted by glycosylation [64,65]. The receptor critical region of CAT1 varies extensively between E-MLV susceptible and resistant species, as well as among susceptible rodent species, none of which has any obvious effect on transport function [66]. Two variants of the laboratory mouse receptor, mCAT1, have been found in wild mice. The *M. dunni* receptor, dCAT1, has an added residue in the receptor critical region (NVKYGGE) and its restriction of MoMLV is regulated by glycosylation [67]. A second variant, found in the African pygmy mouse *M. minutoides,* has a V233L replacement that has no effect on AKV MLV entry [68], although V233 has been assigned a role in virus entry and gp70 binding [69,70]. These two CAT1 variants are carried by mice that have had no known exposure to E-MLVs.

Few CAT1 genes carried by *M. musculus* had been characterized [49], so we examined CAT1 sequences from *M. musculus* subspecies, selected from widely separated geographic locations, including mice with and without E-ERVs. We identified no new variants in the CAT1 ECL3. Ten additional CAT1 genes, mined from the genomes of individually trapped *M. m. castaneus* mice, revealed that four are heterozygous for the V233L polymorphism (H12, H25, H24, H28) and two are homozygotes (H14, H26). 

Evaluation of the rodent CAT1 for positive selection included sequences from various *Mus* species and other rodents (Table S3). The phylogenetic tree generated for this analysis showed strong bootstrap support in the vast majority of branches and revealed clustering of CAT1 sequences from the genus *Mus* and the subfamily *Murinae*, with a clear separation of the species in the different rodent suborders (Figure S2). This screen identified 14 sites under positive selection, including a cluster at the C-terminus of ECL3 with two sites, V233 and E237, which are within the patch implicated in virus entry (Figure 4, Table S4). Limiting the analysis to mice and hamsters, which are generally susceptible to E-MLV infection, identifies only five sites under positive selection, none of which is in ECL3. Thus, while two sites within the receptor critical region are under positive selection in the broader set of rodent species, sites important for E-XRV entry have remained unchanged in taxa exposed to virus challenge. 

This analysis of E-MLVs and their CAT1 receptor in wild mice shows that, although E-MLVs are recently acquired and show substantial variation in *env*, their acquisition, spread, and rapid diversification has not altered the receptor interface in *M. musculus*. While this near absence of CAT1 polymorphism, in response to virus challenge, may reflect a relatively short evolutionary timeframe, a comparable time period produced multiple functional variants of the receptor used by the X/P-MLVs carried by these same mice (see below). One possible explanation for the failure of CAT1 to evolve in response to potentially lethal virus challenge is the presence of the *Fv4* restriction factor in many E-MLV infected mouse populations (Figure 2). The Cas/Frg *env* inserted at this gene produces an Env glycoprotein, originally proposed to block exogenous infection by interference [38], and also has a fusion defect, so its incorporation into virions in virus-infected *Fv4*-positive mice results in entry defective virions [71]. The presence of this dispersed and highly effective *Fv4* antiviral gene, in E-MLV infected wild mice, would thus decrease virus-directed selection pressure on CAT1. This also supports the suggestion that the extensive E-MLV *env* variation is unrelated to receptor interactions.

### 3.2. X/P-MLVs and Their XPR1 Receptor

The XPR1-dependent X/P-XRVs were initially distinguished as two host range groups: broad host range xenotropic XRVs (X-XRVs), otherwise unable to infect cells of most laboratory mice [72], and polytropic XRVs (P-XRVs) able to infect mice and other species [73]. These host range differences are due to polymorphisms in the viral *env* RBD and corresponding changes in the receptor determining regions of XPR1 [11].

#### 3.2.1. XPR1

The functional variants and adaptive evolution of XPR1 have been described previously. To summarize: The X/P-XRVs use the XPR1 receptor to infect cells of *M. musculus* subspecies as well as all but a few other mammals [74]. XPR1 is a ubiquitously expressed phosphate exporter, and sequence variations in phylogenetically distinct species maintain exporter function [75]. *Mus* taxa carry six distinctive functional variants of *Xpr1* [11], one of which, *Xpr1^sxv^,* is permissive, while the rest restrict different subsets of XPR1-dependent viruses. The permissive receptor, *Xpr1^sxv^*, predates the divergence of the house mouse subspecies and their acquisition of MLVs, and is retained exclusively by one subspecies, *M. m. domesticus* [11]. The five restrictive XPR1 receptor variants show a taxon-delimited distribution in Eurasian subspecies: *M. m. musculus* (*Xpr1^m^*), *M. m. castaneus* (*Xpr1^c^*, *Xpr1^c^*^2^), *and M. m. molossinus* (*Xpr1^m^*, *Xpr1^n^*) [76]. These variants are marked by alterations in two of the four putative XPR1 ECLs and include replacement mutations as well as three different but overlapping deletions [11,77]. Additional sequence variants found in *M. tenellus*, and in Iranian *M. musculus*, have not been tested for receptor function [11,14]. Phylogenetic analysis of the rodent *Xpr1* showed it to be under positive selection, affecting key residues implicated in receptor function in ECL3 and ECL4 [11] (Figure 4).

#### 3.2.2. X/P-MLVs

**Figure 5:**
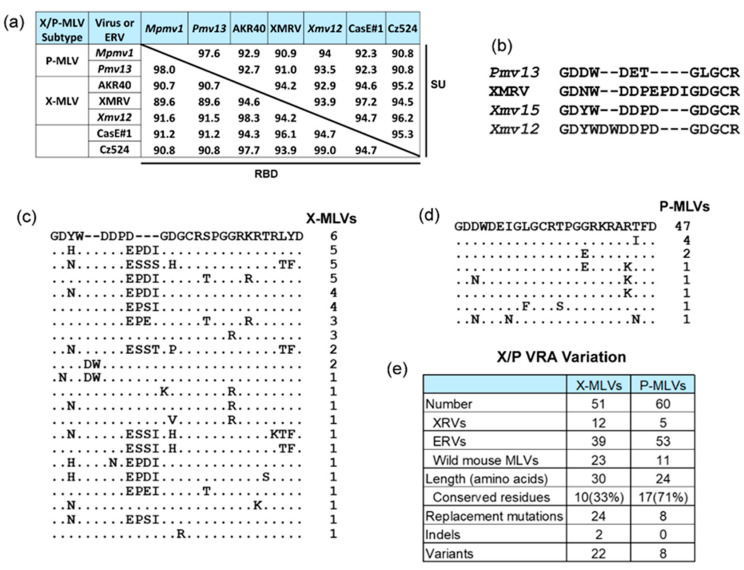
Variation in X/P-MLV *env* genes. (**a**) Matrix showing percent sequence identity of representative X/P-MLVs in SU and RBD. (**b**) Protein alignment of the N-terminal end of VRAenv for a P-ERV and three representative X-MLVs identifying three indels. (**c**,**d**) Differences in VRA*env* sequence variation in X- and P-MLVs and the number of individual MLVs with each variation. (**e**) Summary of the extent of sequence variation in these two sets of MLVs.

Compared to the E-MLVs, X/P-MLVs show considerably less overall sequence divergence in SU and RBD (>89.5%) (Figure 5a), but at least six X-XRV isolates have been described that differ in their ability to use the restrictive XPR1 variants [78]. While the critical Env residues involved in XPR1 receptor interactions have not been identified, receptor choice has been mapped to the VRA variable domain in the RBD [37]. The VRA differs in sequence and size among infectious X/P-XRVs; relative to P-XRVs, the X-XRV RBD*env* is larger, with three distinct indels (insertions/deletions) involving nine codons at the 5′ end of VRA (Figure 5b). Infectious X/P-XRVs with different VRA indel patterns show different XPR1 receptor preferences, suggesting these indels may contribute to receptor specificity [49,78].

Southern blotting previously showed that, unlike E-ERVs, X/P-ERVs are carried by all *M. musculus* subspecies, indicating that X/P-ERVs were acquired by this species earlier than the E-ERVs [5]. Sequenced X/P-ERVs from geographically separated *Mus* taxa identified P-MLV-like VRA indel patterns and overall sequence homologies in Western Europe (*M. m. domesticus*) and in *M. spretus*, which is sympatric with *M. m. domesticus* in Spain and Morocco; these species are partially interfertile, explaining the acquisition of P-MLV ERVs by *M. spretus*. X-ERVs are not detected in *M. m. domesticus* but predominate in the three Eurasian subspecies. The geographic distribution of subspecific *Xpr1* variants coincides with subspecies ranges and with the X/P-MLV *env* subtypes they carry, as previously shown [11].

In addition to their differences in geographic distribution, the P-MLV VRAs show more limited sequence variation compared to the X-MLVs (Figure 5c,d). In contrast to the P-MLVs, the X-MLV VRAs are marked by many more replacement mutations and some indels. This indicates that the conserved receptor/virus interface involving P-MLVs and their permissive XPR1 receptor does not show evidence of genetic conflicts, whereas there is substantial and coordinated sequence and functional variation at the interacting interface of the X-MLVs and their XPR1 receptors in Eurasian subspecies. These different patterns define geographically separated coevolutionary “hotspots” experiencing mutual antagonistic selection and “coldspots” with no evidence of selective adaptations. The existence of such “hotspots” and “coldspots” is one of the key predictions of Thompson’s Geographic Mosaic Theory of Coevolution [12].

The absence of adaptive changes at this interface in *M. m. domesticus* is likely due to the fact that P-ERVs do not produce infectious virus or transmit without the assistance of replication competent XRVs, which this subspecies does not harbor [5]. Infectious P-XRVs result from recombination [79], and transcribed products of P-ERVs can co-package into E-XRV virions [80,81]. These P-ERVs therefore pose less of a risk to their hosts than the ERVs carried by Eurasian mice, which can produce infectious X-, as well as E-XRVs [82,83].

Newly sequenced X/P-MLV *env* genes from geographically separated *Mus* taxa were aligned with known X/P-MLVs to construct phylogenetic trees using the full length *env* and a set of RBD sequences. Both trees showed strong bootstrap support at the majority of branches and a clear separation of X-MLVs and P-MLVs was observed (Figures S3 and S4). These sequences were evaluated for evidence of positive selection and together revealed 18 such sites in the RBD (Table S4). The one site identified by all five programs in both DNA sets is 217T, which maps near the C-terminus of the RBD and is one of two adjacent sites with a key role in mediating X/P-XRV entry into human and mink cells [84]. Six positively selected sites were mapped to the VRA and form a patch overlapping the six adjacent residues involved in the various deletions associated with the different entry phenotypes (Figure 4 and Table S4). Three additional sites under positive selection map to the second major variable region in RBD, VRB, which has a secondary role in virus entry [37].

Just as the domesticated *Fv4* E-ERV *env* blocks replication of E-XRVs, there is evidence of comparable X/P-ERV *env* genes that serve protective functions. The first of these, *Rmcf*, has only been found in DBA/2 and related inbred strains [85,86], but *M. m. castaneus* carries at least one similar gene, *Rmcf2*, and possibly others [74,87]. While the CAT1 receptor may have been protected by *Fv4* from selective pressures exerted by E-MLV infection, the distribution of *Rmcf*-like genes in wild mice has not been determined, but it is clearly not significant enough to obviate the co-adaptive changes that have altered XPR1 and X/P-MLVs.

### 3.3. Fv1 and Its Capsid Target

#### 3.3.1. *Fv1*

The first antiretroviral host factor to be discovered was *Fv1* [88]. *Fv1* is a rodent-specific restriction factor that was identified for its post-entry restriction of different subgroups of mouse-tropic MLVs [89], but it can also restrict other retroviruses [90]. *Fv1* is a co-opted retrovirus-related capsid sequence derived from the ERV-L family [91,92], and while it was originally thought to have originated in *Mus*, it was subsequently identified in other rodents [93,94]. The *Fv1^n^* allele, first identified in NIH Swiss cells, limits the replication of B-tropic E-XRVs, and the *b* allele, found in BALB/c mice, restricts N-tropic E-XRVs [95]. Laboratory strains carry two other restriction variants (*Fv1^nr^*, *Fv1^d^*) [96,97], and there are additional sequence and functional variants in wild mice [10]. These restriction variants are distinguished from one another at four residues in the C-terminal half of the gene, and by variations in length and sequence at the C-terminus, all of which influence the restriction phenotype [98]. The *Mus Fv1* shows positive selection that is centered on six codons including three of the seven residues known to govern restriction (261, 268, 270, 349, 352, 358, 399) [90,98] (Figure 4). Additional residues under positive selection were identified in the expanded set of rodents carrying *Fv1* [93,94]. 

**Figure 6:**
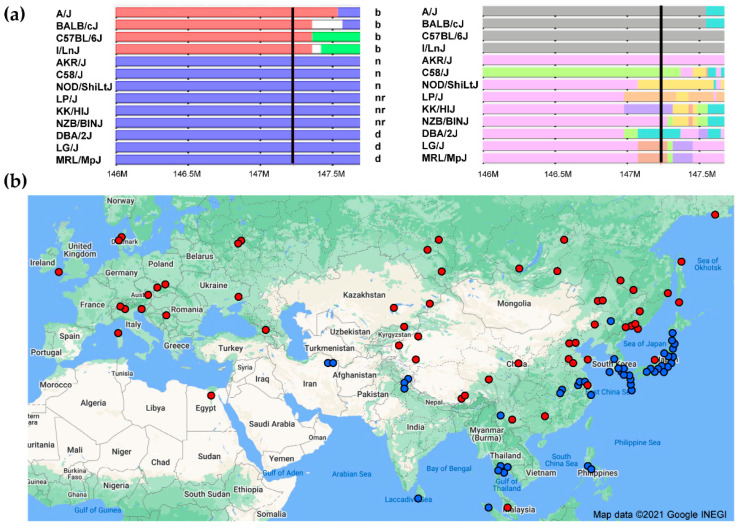
Distribution of *Fv1* variants in mouse strains and species. (**a**) *Fv1^b^* is embedded in a segment of Chromosome 4 derived from *M. m. musculus* (Japan). The left panel shows subspecies origins in the chromosome regions surrounding the location of *Fv1* indicated by a black bar. Blue tracks, *M. m. domesticus*; red, *M. m. musculus*. The right panel shows SNP-defined haplotype regions defined by different colors. (**b**) Geographic distribution of *Fv1* variants having C-termini that are *Fv1^b^*-like (blue) or *Fv1^n^*-like (red).

The inbred strains represent different mosaics of the *M. musculus* subspecies [99]. The Mouse Phylogeny Viewer (MPV) is an online tool that identifies the subspecies of origin of segments along each chromosome for 98 inbred strains [4]. Here, we typed 35 mouse strains in the MPV dataset for *Fv1* [42], and Figure 6a shows that the *Fv1^b^* allele originated in Japanese *M. m. musculus*, while the *Fv1^n,nr,d^* alleles are embedded in segments derived from *M. m. domesticus*. These three *M. m. domesticus* derived *Fv1* alleles, however, are not found in shared haplotype segments defined by common flanking SNPs, suggesting their independent derivation or acquisition through mutation or recombination. 

*Fv1^b^* and *Fv1^n,nr,d^* are distinguishable by a 1.3 kb indel at the *Fv1* 3′ terminus that extends *Fv1^b^* by 22 residues [92]. Our PCR analysis of Eurasian mouse DNAs found the *Fv1^b^*-like extension in 19/20 Japanese mice, in mice from SE Asia, and in Iranian mice, identified as *M. bactrianus* (Figure 6b). Mice from northern China, Russia, Eastern and Western Europe all produced the smaller *Fv1^n^*-like segment.

*Fv1* sequences from selected house mice were determined by direct sequencing or were extracted from the genomes of ten *M. m. castaneus* mice. Of the 34 genes, 22 had an *Fv1^b^* extension, one of which included a 4 bp insert (Table 2). The residues that distinguish these genes are at positions 270, 352, 358, and 399; these sites are all functionally relevant, and three are under positive selection in *Mus* (Figure 4) [10]. The K358E substitution, found in *Fv1^b^* laboratory strains, was identified in only one of these genes—in a Japanese mouse. All 12 genes with short C-termini have residue combinations corresponding to three of the inbred strain alleles. Eight of the 22 genes with the longer extension have unusual residue combinations, suggesting these mice might face viral antagonists.

An *Fv1* ORF is present in most other species in the *Mus* genus [10,100], and these have large C-terminal insertions [10], suggesting that this configuration is the ancestral form. Thus, the shorter *Fv1^n^*-like versions of this gene were likely generated by deletion and then acquired mutations at key sites. The retention of *Fv1^b^*-like genes is most prevalent in mice harboring Cas/Frg E-ERVs, which have B-tropic capsid genes that are therefore not restricted by *Fv1^b^*, a clear adaptive advantage for these MLVs.

#### 3.3.2. Viral Capsid Target of *Fv1*

*Fv1* restriction targets the capsid of the viral *gag* gene [101,102]. Sequence comparisons and mutagenesis determined that the site determinative for N/B tropism is 110 [97], and residue replacements at other capsid sites can contribute to alternative restriction patterns defined as NR-tropic [96,103], D-tropic [97], or NB-tropic (viruses insensitive to both *Fv1^n^* and *Fv1^b^*) [104,105] (Figure 4). Virtually all laboratory mouse E-ERVs are N-tropic and carry R110. B-tropic E-MLVs, which carry E110, can be isolated from aging mice of *Fv1^b^* strains [106], and some *Fv1^b^* mice have acquired B-tropic E-MLV ERVs [107]. The C57BL/6 mouse reference genome carries *Fv1^b^* and has one E-ERV, *Emv2*, which is N-tropic and 36 X/P-ERVs with *gag* ORFs, of which 34 have the residue associated with B-tropism, E110.

We used these capsid gene sequences to construct a phylogeny. As shown in Figure S5, this phylogenetic tree showed strong bootstrap support at the majority of the nodes. Nine sites were determined to be under positive selection (Table S4). All programs identified positive selection at position 110, which determines N/B tropism, but at no other site implicated in *Fv1*-determined tropisms; no replacement mutations were found at positions 114 and 117, and the variations found at 92-95 did not show selection. These findings establish that the antagonistic interaction between *Fv1* and its viral target has resulted in coevolutionary adaptive changes at the sites of interaction of both participants in this arms race.

## 4. Conclusions

The mouse gammaretroviruses co-evolved with their host species, and while the recently acquired MLV ERVs can retain the ability to produce viral proteins and infectious viruses, there are many host antiviral restriction genes that interfere with these processes. These interacting agents are subject to bidirectional selective pressures, resulting in a cyclical process that produces viruses that evade host restrictions and counter-adaptive changes to the restriction factors. Another component in this process is the geographic segregation of virus-infected *M. musculus* subspecies resulting in mosaic patterns of adaptations that generate distinctive MLV and restriction factor variants that can be taxon as well as locality specific.

The three virus/host pairings examined here provide examples of different co-evolutionary patterns. Our previous work showed that *Fv1* and the XPR1 receptor are under positive selection in rodents [11,93] and that XPR1 has distinctive functional variants in house mouse subspecies [10,11]. Here, we show that *Fv1* alleles also show defined geographic distributions as well as novel variants in house mouse populations. We also show that the variations in the *Fv1* and XPR1 sites, critical for restriction, correlate with mutations in their viral targets that are also under positive selection.

There are two examples of coevolutionary “cold spots” with no significant selection at the interacting sites. First, the E-MLVs and their CAT1 receptor in *M. musculus* are not polymorphic at their interfaces in any of the three subspecies carrying E-MLVs. While that means these mice should be vulnerable to infection, their survival in the face of virus challenge is at least partly ensured by the presence of the *Fv4* E-ERV in some of these populations. The second example, with no obvious virus-host conflict, is found in *M. m. domesticus* mice that retain the ancestral and fully permissive *Xpr1^sxv^* receptor. These mice carry P-ERVs, not known to produce virus except by recombination with XRVs of other host range groups [79]; P-ERVs can spread but this is XPR1-independent [108]. Thus, there is no ongoing conflict, as *Xpr1* is under no pressure to adapt to ERVs that do not produce infectious virus, and variation in the P-ERV VRAs is much more limited than in their X-ERV counterparts.

Adaptations that provide a clear survival advantage to their hosts can spread rapidly through populations and into neighboring populations. *Fv1* and *Fv4* are both ERVs that have been coopted for antiviral functions; *Fv1* is the *gag* capsid gene of the ancient MuERV-L family, while *Fv4* is a Cas/Frg *env*. We show that *Fv4* likely arose in SE Asia, but it is now fairly widespread in Japan and is also found in pockets of California [5]. For *Fv1*, *b*-like variants predominate in Japan and are present in about half the *M. m. castaneus* population in SE Asia and through southern Asia to Iran, which is the ancestral home of *M. musculus* [6]. *Fv1^b^*-like genes are also found in basal species in the genus *Mus* [10] suggesting that *Fv1^b^* is the ancestral form, and the *Fv1^n^* alleles were generated by deletion. Most wild mouse ERV *gag* genes carry the B-tropic determinant and are therefore not restricted by the *Fv1^b^* gene prevalent in these mice.

ERVs are stable components of the mammalian genome that can alter host gene function and that can induce a variety of pathologies. Analysis of these antagonistic pairings of restrictive genes and viruses has enhanced our understanding of their origins, spread, and diversification. These entities co-evolve on a temporal scale, as evidenced by positive selection at their interacting interfaces that produces functional variants, but their evolution can also be understood on a spatial scale with distinctive sets of virus/host combinations in different locations, as predicted by the Geographic Mosaic Theory of Evolution [12].

## Figures and Tables

**Table 1:** Phenotypic changes in viruses, with replacement mutations, at receptor critical sites.

Polymorphisms at Receptor Critical Sites	MLV or ERV	Phenotype	Reference
S76Δ,S77Δ	MoMLV	Reduces infection of *M. dunni* cells	[52]
S84A	F-S MLV	Induces syncytia in *M. dunni*, infects hamster cells	[51]
	Cas/Frg E-ERVs	Unknown	
S82F ^1^	Mo-Spl574	Induces syncytia in *M. dunni*, restricted in other mouse cells	[53]
S82F, E114G ^1^	Spl574-E114G	Correction of host range restriction of Spl574	[54]
W102G	TR1.3 (FrMLV)	Syncytia formation in SC-1 cells, neurologic disease	[55]
E116G, W129K	PVC-211 (FrMLV)	Enhanced ability to infect hamster cells	[56]

^1^ Numbering in MoMLV variants reflects the two codon upstream deletion.

**Table 2:** Restriction critical residues in sequenced wild mouse *Fv1* genes.

		*Fv1* Sites				
*Fv1* Type ^1^	Subspecies, Name (Location)	270	352	358	399	C-Terminus ^2^
*n* or *n*-like	*domesticus*, SK/Cam (UK)		S	K	V	n
	*domesticus*, CalWM (USA)	K	S	K	V	n
	*musculus*, PWK (Czech Republic)	K	S	K	V	n
	*musculus*, SKIVE (Denmark)	K	S	K	V	n
*nr* or *nr*-like	*musculus*, (Novobirsk, Russia)		F	K	V	n
	*musculus*, CZII (Slovakia)	K	F	K	V	n
	spp., Aks (China)	K	F	K	V	n
	spp., Las (China)	K	F	K	V	n
	spp., (Vladivostok, Russia)	K	F	K	V	n
	*domesticus*, ZALENDE (Switzerland		F	K	V	n
	*domesticus*, CLA (USA)		F	K	V	n
*d*-like	*domesticus*, PRAE (Morocco)	Q	S	K	V	n
*b* or *b*-like	*molossinus*, MOM (Japan)	K	S	E	R	b
	*bactrianus*, (Iran)		S	K	V	b
	*castaneus*, (Philippines)		S	K	V	b
	*castaneus*, H12 (India) ^5^	K/Q ^3^	S	K	V	b/b2 ^3^
	*castaneus*, H30,34 (India) ^5^	K/Q ^3^	S	K	V	b3
	*castaneus*, H15,27 (India) ^5^	K/R ^3^	S	K	V	b
	*castaneus*, H24,26,28,36 (India) ^5^	K	S	K	V	b
	*molossinus*, MOLD (Japan)	K	S	K	R	b
	*molossinus*, MOLC (Japan)	K	F	K	R	b
	*molossinus*, MAE (Japan)	K	F	K	R	b
	*molossinus*, JF1/Ms (Japanese fancy mouse)	K	F	K	R	b
	*molossinus*, (Saitama, Japan)		F	K	R	b
	spp., IAS3 (Korea)		F	K	R	b
	spp., Wuh (China)		S	K	R	b
	*domesticus* LEWES (USA)	Q	S	K	V	b3
	*castaneus*, CAST/EiJ (Thailand)	Q	S	K	V	b4
	*castaneus*, H14 (India) ^5^	Q	- ^4^	- ^4^	- ^4^	- ^4^

^1^ Based on C-terminus for *b*, 352 for *n* and *nr*, 270 for *d*. ^2^ C-terminus types: n, ELSLKPTAATKL; b, ELSLKPTAAGLTSVGSVGVLSLSPWKHQSNS; b2, ELSLKPTAAGLTAGLAPVGSVGVLSLSPWKHQSNS; b3, ELRGQR; b4, ELSLKPTAAGLPAGLASVGSVGVLSLSPWHKH. ^3^ Heterozygous. ^4^ Stop gain variant at position 327 (TGG > TAG) in both H14 alleles, resulting in predicted protein truncation or lack of expression of the corresponding allele. This variant has the b2 C-terminus. ^5^ The ten *Fv1^b^*-like *castaneus* H genes are identical to *Fv1^b^* except where indicated and a 137T/S substitution in H34.

## Data Availability

Data is contained within the article or supplementary material.

## Supplementary Materials

The following are available online at https://www.mdpi.com/article/10.3390/v13091864/s1, Datafile S1: Additional sequences, Figure S1: Phylogeny of E-MLV env sequences, Figure S2: Phylogeny of CAT1/*Slc7a1* in rodents, Figure S3. Phylogeny of X/P-MLV env RBD sequences, Figure S4. Phylogeny of X/P-MLV env sequences, Figure S5. Phylogeny of X/P and E-MLV capsid sequences, Table S1. Sources of wild-caught and wild-derived mice and mouse DNAs, Table S2. Primer sequences used for PCR, Table S3. Accession numbers for MLV XRVs and ERVs and for *CAT1* and *Fv1* gene sequences, Table S4. Residues under positive selection as identified by separate programs.
