# Supplementary material for: Patterns of Coevolutionary Adaptations across Time and Space in Mouse Gammaretroviruses and Three Restrictive Host Factors
Source: Viruses. 2021 Sep 18;13(9):1864. doi: 10.3390/v13091864 (PMC8472935; doi:10.3390/v13091864)
Supplement: Supplementary file 1 [file viruses-13-01864-s001.zip › Supplementary_Information_Revised.pdf]

Supplementary Information for

Patterns of coevolutionary adaptations across time and space in mouse gammaretroviruses and three restrictive host factors

Guney Boso, Oscar Lam, Devinka Bamunusinghe, Andrew J. Oler, Kurt Wollenberg, Qingping Liu, Esther Shaffer and Christine A. Kozak

Datafile S1. Additional sequences (Provided as a separate text file)

Supplementary Figures

Figure S1. Phylogeny of E-MLV *env* sequences. Full-length envelope gene sequences of the indicated E-MLVs were aligned and a maximum likelihood tree was generated using RaxML with 500 replicates. Bootstrap values are shown at each node. The tree was midrooted.

Figure S2. Phylogeny of *Cat1/Slc7a1* in rodents. *Cat1/Slc7a1* coding sequence of the indicated rodent species were aligned and a maximum likelihood tree was generated using RaxML with 500 replicates. Bootstrap values are shown at each node. The tree was rooted at Rabbit *Slc7a1* sequence.

Figure S3. Phylogeny of X/P-MLV *env* RBD sequences. Sequences corresponding to the RBD of the *env* gene of the indicated X/P-MLVs were aligned and a maximum

likelihood tree was generated using RaxML with 500 replicates. Bootstrap values are shown at each node. The tree was midrooted.

Figure S4. Phylogeny of X/P-MLV *env* sequences. Envelope gene sequence of the indicated X/P-MLVs were aligned and a maximum likelihood tree was generated using RaxML with 500 replicates. Bootstrap values are shown at each node. The tree was midrooted.

Figure S5. Phylogeny of X/P and E-MLV capsid sequences. Sequences corresponding to the capsid domain of the *gag* gene sequence of the indicated X/P and E-MLVs were aligned and a maximum likelihood tree was generated using RaxML with 500 replicates. Bootstrap values are shown at each node. The tree was midrooted.

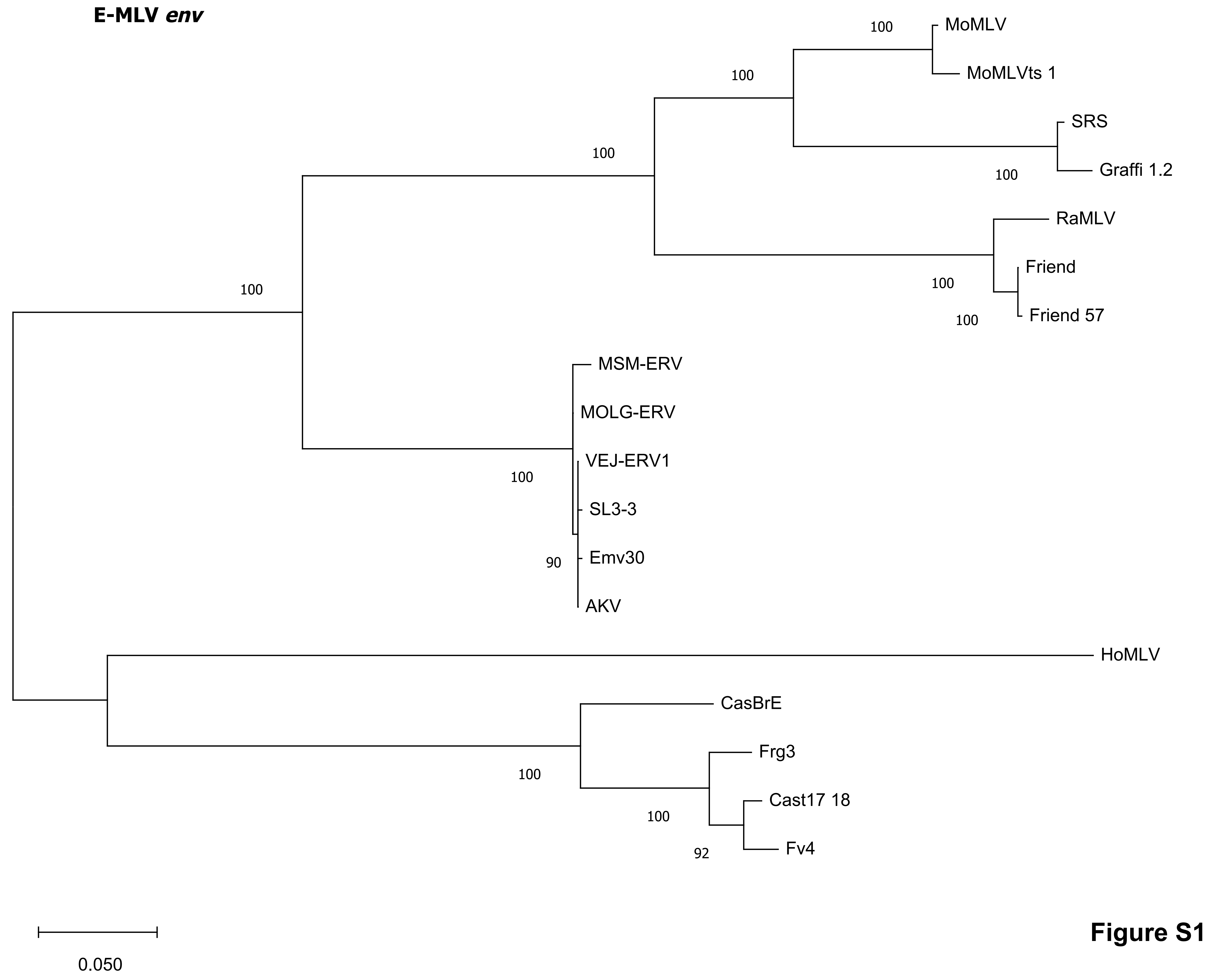
E-MLV *env*
MoMLV
MoMLVts 1
SRS
Graffi 1.2
RaMLV
Friend
Friend 57
MSM-ERV
MOLG-ERV
VEJ-ERV1
SL3-3
Emv30
AKV
HoMLV
CasBrE
Frg3
Cast17 18
Fv4
100
100
100
100
100
100
100
100
90
100
100
92
0.050

Figure S1

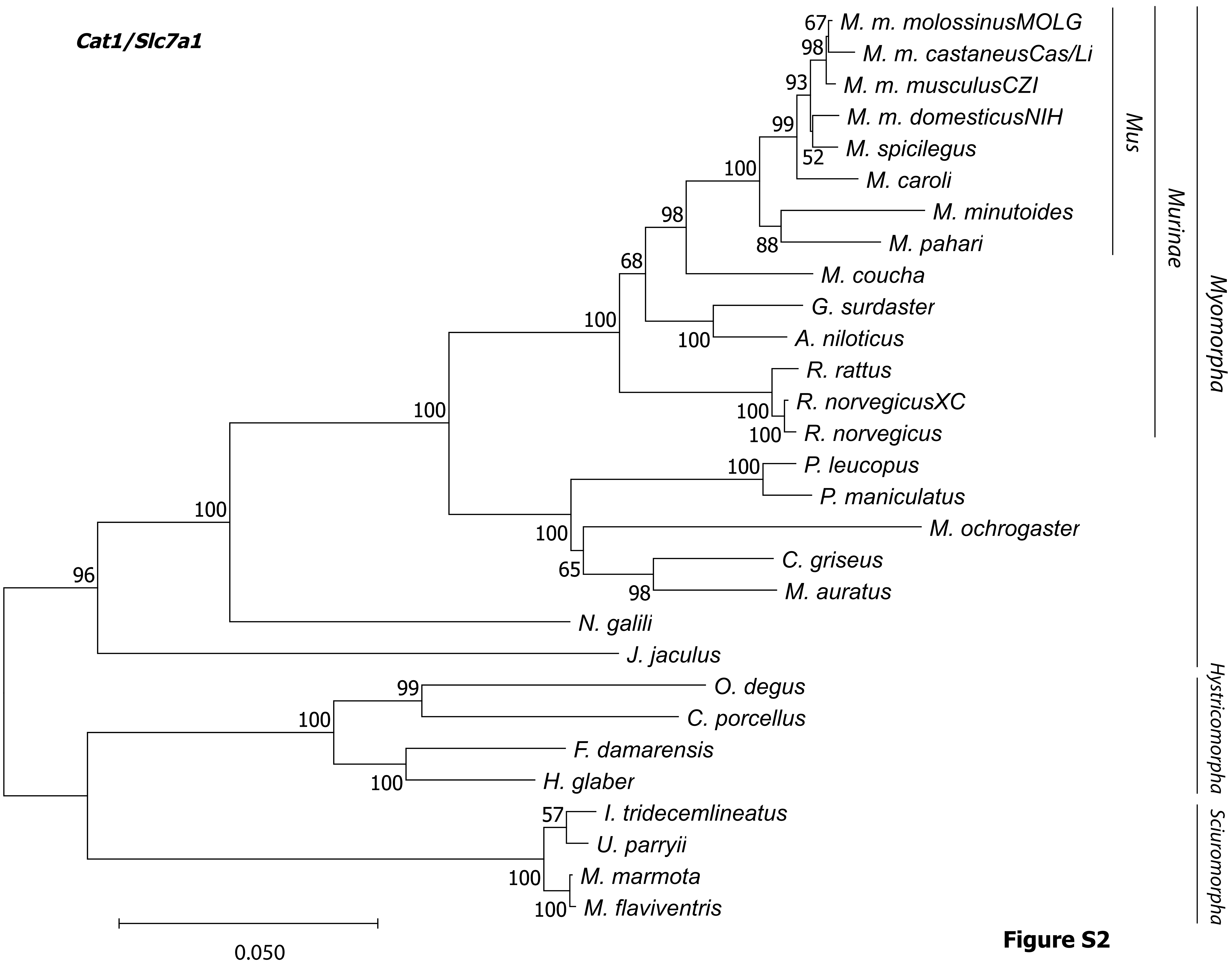

Cat1/Slc7a1
M. m. molossinusMOLG
M. m. castaneusCas/Li
M. m. musculusCZI
M. m. domesticusNIH
M. spicilegus
M. caroli
M. minutoides
M. pahari
M. coucha
G. surdaster
A. niloticus
R. rattus
R. norvegicusXC
R. norvegicus
P. leucopus
P. maniculatus
M. ochrogaster
C. griseus
M. auratus
N. galili
J. jaculus
O. degus
C. porcellus
F. damarensis
H. glaber
I. tridecemlineatus
U. parryii
M. marmota
M. flaviventris
67
98
93
99
52
100
98
88
68
100
100
100
100
100
100
100
100
65
98
100
96
99
100
100
57
100
100
Mus
Murinae
Myomorpha
Hystricomorpha
Sciuromorpha
0.050

Figure S2

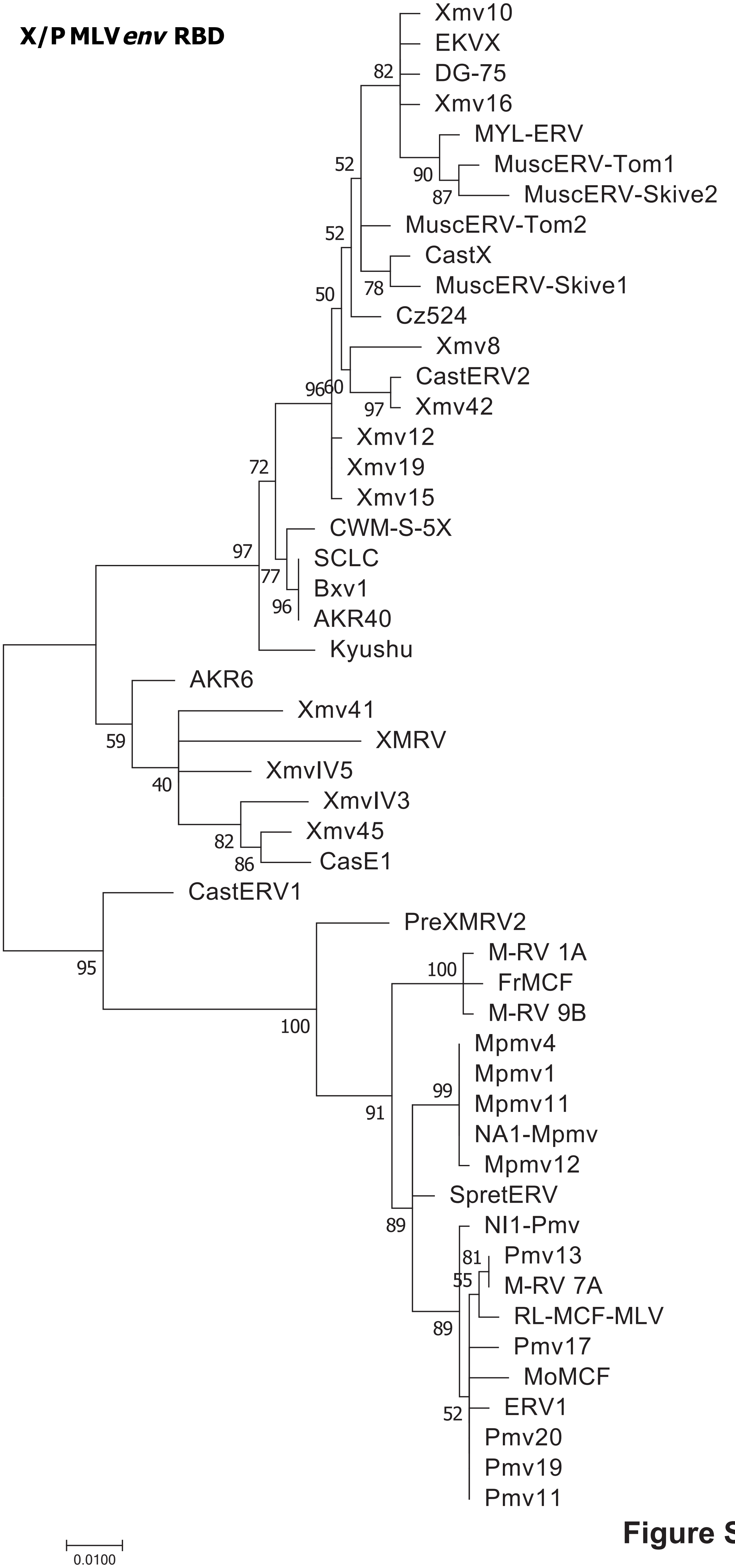

X/P MLV *env* RBD
Xmv10
EKVX
DG-75
Xmv16
MYL-ERV
MuscERV-Tom1
MuscERV-Skive2
MuscERV-Tom2
CastX
MuscERV-Skive1
Cz524
Xmv8
CastERV2
Xmv42
Xmv12
Xmv19
Xmv15
CWM-S-5X
SCLC
Bxv1
AKR40
Kyushu
AKR6
Xmv41
XMRV
XmvIV5
XmvIV3
Xmv45
CasE1
CastERV1
PreXMRV2
M-RV 1A
FrMCF
M-RV 9B
Mpmv4
Mpmv1
Mpmv11
NA1-Mpmv
Mpmv12
SpretERV
NI1-Pmv
Pmv13
M-RV 7A
RL-MCF-MLV
Pmv17
MoMCF
ERV1
Pmv20
Pmv19
Pmv11
0.0100

Figure S3

X/P MLV*env*

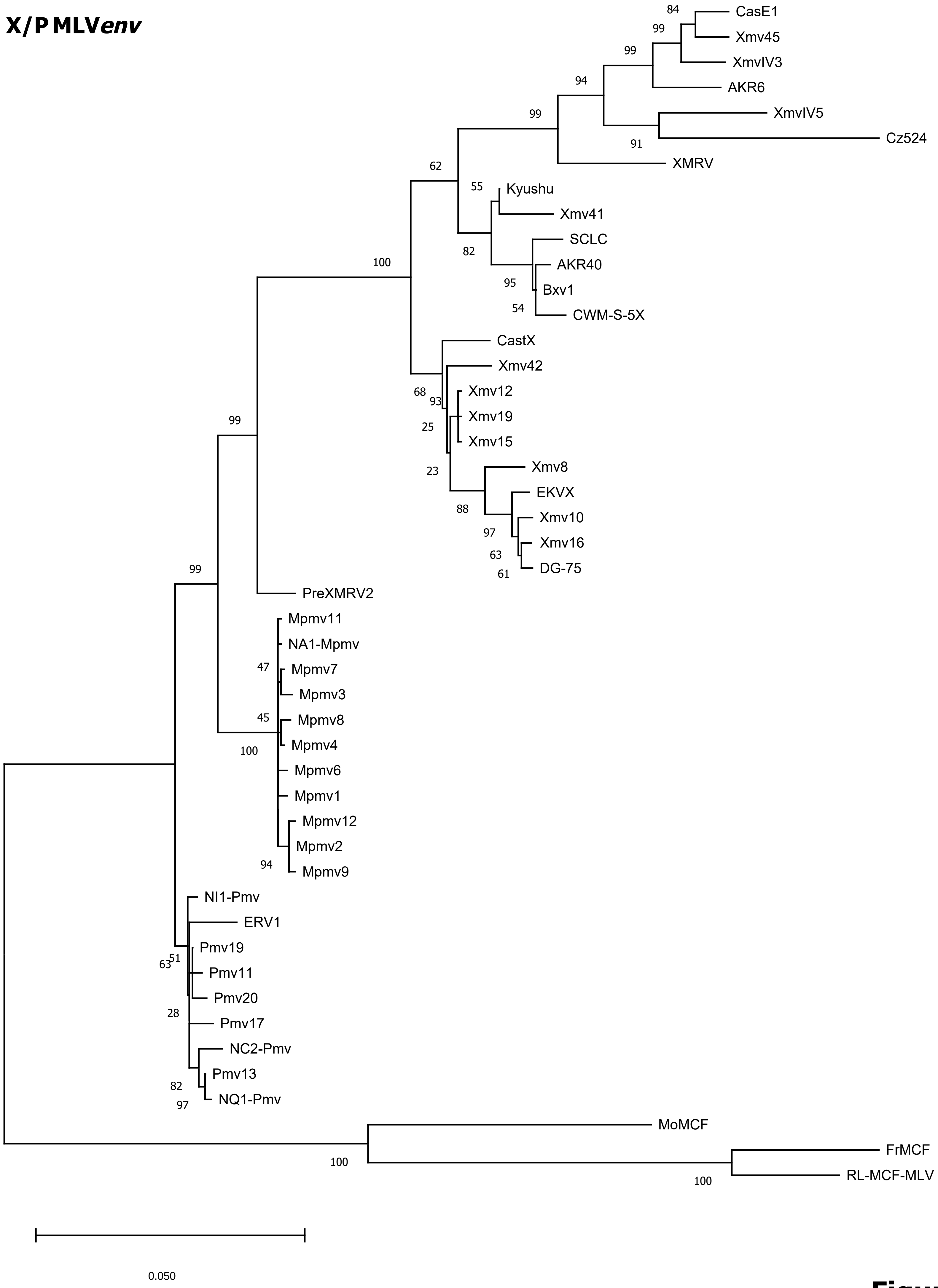

Figure S4

X/P and E-MLV capsid

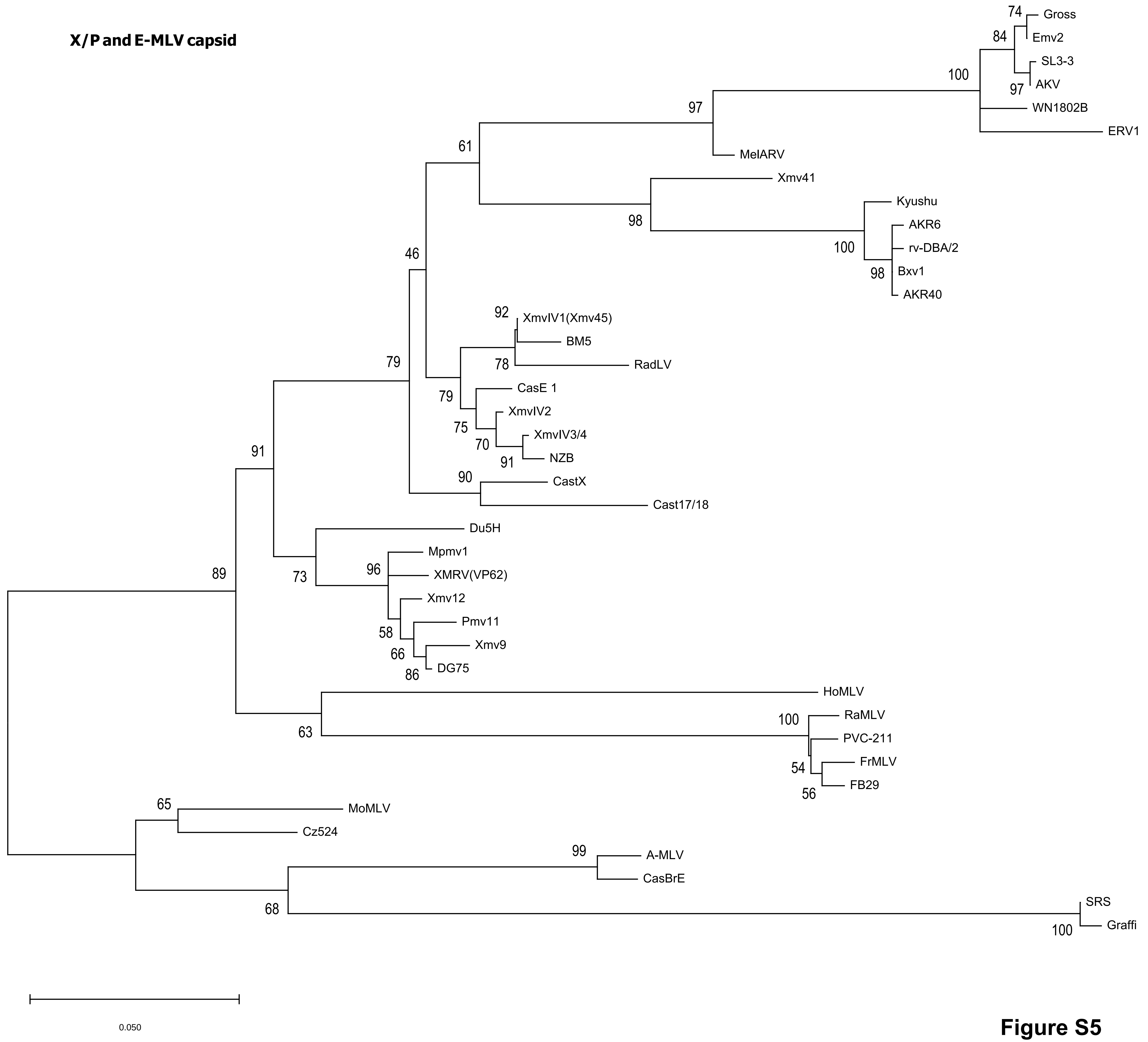

Figure S5

Supplementary Tables

Table S1. Sources of wild-caught and wild-derived mice and mouse DNAs.

Mus musculus subspecies[1]	Alternate designation	Trapping location	DNA ID #	Source	Used in[2]	
					Figure 2	Figure 6b
bactrianus	M.Bac-Iran	Mashhad, Iran	MG-0417 (1510)	RIKEN	X	X
bactrianus	M.Bac-Iran	Mashhad, Iran	MG-5047 (1622)	RIKEN		X
castaneus	Bal	Bandar, Sumatra, Indonesia	MG-5121 (111)	RIKEN	X	X
castaneus	CASA/RkJ	Thailand		Jackson	X	X
castaneus	CAST/17	Pathum Thani, Thailand		Hartley	X	X
castaneus	CAST/N	Thonburi, Thailand		Potter	X	
castaneus	CAST/EiJ	Thonburi, Thailand		Jackson		X
castaneus	CAST/Rp	Thonburi, Thailand		Roswell Park		
castaneus	CAS/Li	Thonburi, Thailand		Potter		X
castaneus	CASP/1Nga			RIKEN	X	X
castaneus	H14,H12	Katrain, India		Halligan/ Keightley		X
castaneus	H24,H15,H26 ,H27	Mandi, India		Halligan/ Keightley		X
castaneus	H30,H28,H34 ,H36	Kunihar, India		Halligan/ Keightley		X
castaneus	HMI/Ms	Hemei, Taiwan		RIKEN,Ab e	X	
castaneus	Mal	Malaysia	MG-5060 (1635)	RIKEN	X	X
castaneus	MYS/Mz			RIKEN		
castaneus	Nin	Ningpo, China	MG-0795 (259)	RIKEN	X	X
castaneus	Qzn	Quezon City, Philippines	MG-0421 (1514)	RIKEN	X	X
castaneus	M.mus-sp	Vladivostok, Kirova 62, U.S.S.R.	MG-3077 (195)	RIKEN	X	X
domesticus	ABUR	Abu Rawash, Egypt		Potter	X	X
domesticus	BIBB	Sede Boger, Israel	MG-0377 (1474)	RIKEN	X	
domesticus	BQC	Bouquet Canyon, CA		Potter		X

domesticus	CALB/RkJ	California		Jackson		
domesticus	CalWM (7)	Lake Casitas, CA		Rasheed		
domesticus	CLA (Centreville Lite)	Centreville, MD		Potter		
domesticus	DFC	Corse, France	MG-0385 (1481)	RIKEN		X
domesticus	HAF (Havens Farm)	Davidsonville, MD		Potter		
domesticus	JJD (J.J.Downs)	Ridgely, MD		Potter		
domesticus	LW, LEWES/EiJ	Lewes, DE		Potter, Jackson		
domesticus	PERA/EiJ	Rimac Valley, Peru		Jackson		
domesticus	PERC/EiJ	Rimac Valley, Peru		Jackson		
domesticus	PGN2	Pegion region, Canada		Abe		
domesticus	TIRANO, poschiavinus	Tirano, Italy		Potter	X	X
domesticus	ZALENDE, poschiavinus	Zalende, Switzerland		Potter	X	X
domesticus	PRAE, praetextus (PRAE)	Erfoud, Morocco		Potter	X	
domesticus	SAF (Sanners Farm)	Davidsonville, MD		Potter		
domesticus	SC-1 cells	California		Hartley		
domesticus	SF/CamEiJ	Marin County, CA		Jackson		
domesticus	SK/Cam	Skokholm Is., U.K.	MG-0067 (1294)	RIKEN	X	X
domesticus	WS (Watkins Star)	Watkins Farm, MD		Potter		
domesticus	WMP/PasDnJ	Monastir, Tunisia		Jackson	X	
M.m.gansuensis spp.	Htn	Hotan, China	MG-0762 (226)	RIKEN	X	X
M.m.gansuensis	M.sub-Aks	Akus, China	MG-0608 (4)	RIKEN	X	X
M.m.gansuensis	M.sub-Kes	Kashi, Chaina	MG-0686 (60)	RIKEN	X	X
M.m.homourus	M.sub-Jin	Jinan, China	MG-0784 (248)	RIKEN	X	X
M.m.homourus	M.sub-Zhe	Zheng Zhou, China	MG-0928 (647)	RIKEN	X	X
Mus.leggada	Leg-Per	Peradeniya, Sri Lanka	MG-0273 (1382)	RIKEN		X
molossinus	Aiz1	Aizuwakamatsu, Fukushima, Japan	MG-0489 (1575)	RIKEN	X	X

molossinus	Hkz	Hakozaki, Fukuoka, Japan	MG-0461 (1551)	RIKEN	X	X
molossinus	JF1			Jackson		
molossinus	Kgs	Kagoshima, Kagoshima, Japan	MG-5013 (1593)	RIKEN	X	X
molossinus	Kor	Kohriyama, Fukushima, Japan	MG-0492 (1578)	RIKEN	X	X
molossinus	Mol-KOR1/Stm	Koriyama, Japan		RIKEN		
molossinus	KOR5	Koriyama, Fukushima, Japan		RIKEN	X	
molossinus	KOR7	Kouriyama, Fukushima, Japan		RIKEN		
molossinus	MAE	Maesawa, Iwate, Japan		RIKEN	X	
molossinus	Mae2	Maesawa, Iwate, Japan	MG-0494 (1580)	RIKEN	X	X
molossinus	Mol/Li	Kyushu, Japan		Potter		
molossinus	MOLD/RkJ, MOLF/EiJ, MOLG/DnJ, MOLC/Rk	Fukuoka, Kyushu, Japan		Jackson	X	X
molossinus	MOM	Mizuho Aichi, Japan		RIKEN	X	
molossinus	Mro	Morioka, Iwate	MG-0271 (1380)	RIKEN	X	X
molossinus	MSM	Mishima, Shizuoka, Japan	MG-0082 (1297)	RIKEN,Abe	X	X
molossinus	MZH (MOM)	Mizuho, Aichi, Japan	MG-0099 (1310)	RIKEN	X	X
molossinus	Nig	Niigata, Japan	MG-0202 (1324)	RIKEN	X	X
molossinus	Ohm	Oma, Aomori, Japan	MG-0282 (1389)	RIKEN	X	X
molossinus	STM1,STM2	Japan		RIKEN		
molossinus	Tsm	Tsushima, Nagasaki, Japan	MG-0441 (1532)	RIKEN	X	X
molossinus		Ashiro, Iwate, Japan	MG-0257 (1370)	RIKEN	X	X
molossinus		Higashiohmiya, Saitama, Japan	MG-0210 (1332)	RIKEN	X	X
molossinus		Kanazawa, Ishikawa, Japan	MG-0255 (1368)	RIKEN	X	X
molossinus		Kyoto, Japan	MG-0422 (1515)	RIKEN	X	X
molossinus		Mito, Ibaragi, Japan	MG-0235 (1357)	RIKEN	X	X
molossinus		Nirayama, Shizuoka, Japan	MG-0219 (1341)	RIKEN	X	X

molossinus		Ohmiya, Saitama, Japan	MG-0335 (1436)	RIKEN	X	X
molossinus		Shizuoka, Shizuoka, Japan	MG-0427 (1520)	RIKEN	X	X
molossinus		Takatsuki, Osaka, Japan	MG-0240 (1362)	RIKEN	X	X
molossinus		Tanegashima, Kagoshima, Japan	MG-0442 (1533)	RIKEN	X	X
musculus	Akt/TUA	Aktyubinsk, Kazakhstan		RIKEN	X	
musculus	Ast/TUA (wagneri)	Astrakhan, Russia		RIKEN	X	
musculus	BLG2	Toshevo, Bulgaria		Abe	X	
musculus	CZI, CZECH/EiJ	Morovia, Czech Republic		Potter	X	X
musculus	CzII, CZECHII/EiJ	Bratislava, Slovakia		Potter	X	X
musculus	Gor/TUA	Gorno-Altaisk, Russia		RIKEN	X	X
musculus	Hul	Hulin, China	MG-0920 (639)	RIKEN	X	X
musculus	IRK/TUA	Irkutsk, E. Siberia, Russia		RIKEN	X	X
musculus	Jix	Ji Xian, China	MG-0843 (562)	RIKEN	X	X
musculus	KAZ/TUA	Alma-Ata, Kazakhstan		RIKEN	X	X
musculus	Krk1	Krakow, Poland	MG-5235 (1766)	RIKEN	X	X
musculus	MBT/Pas	Toshevo, Bulgaria		RIKEN		
musculus	MYL	Ljubljana Slovenia, Yugoslavia	MG-0399 (1494)	RIKEN	X	X
musculus	NJL	Northern Jutland, Denmark		Abe,RIKEN	X	
musculus	PWD/PhJ	Kunratice, Czech Republic		Jackson	X	X
musculus	PWK/PhJ	Lhotka, Czech Republic		Jackson		X
musculus	Skive	Skive, Denmark		Potter	X	X
musculus	Tom/TUA	Tomsk,Tuva, Siberia, USSR, Russia		RIKEN	X	X
musculus	Okh/TUA	Okha, Russia		RIKEN	X	X
musculus	VEJ	Vejrumbro, Denmark		Potter,Morse	X	X
musculus		Belgrade		Morse		X
musculus		Brno		Morse	X	
musculus		Viborg		Morse		X

tantillus	Las	Lasa, China	MG-0723 (90)	RIKEN	X	X
musculus spp. M.wag-Ton	m.wag-Ton	Tongliac, China	MG-0856 (575)	RIKEN	X	X
musculus (wagneri)	KNB/TUA	Balkhash Lake, Kazakhstan		RIKEN	X	X
spp.	Bjn2	Beijing, China	MG-5066 (2009)	RIKEN	X	X
spp.	Dal	Dali, China	MG-0788 (252)	RIKEN	X	X
spp.	Gui	Guilin China	MG-0501 (1876)	RIKEN	X	
spp.	Gui	Guilin, China	MG-0502 (1877)	RIKEN		X
spp.	Ias3	Suweon, Korea	MG-5018 (1598)	RIKEN	X	X
spp.	Jia	Jiangyin, China	MG-2100 (842)	RIKEN	X	X
spp.	Jin	Jinan, China	MG-0790	RIKEN		X
spp.	Kjr	Kojuri, Korea	MG-0060 (1290)	RIKEN	X	X
spp.	Kun	Kunming, China	MG-0529	RIKEN	X	X
spp.	Lai	Laiyang, China	MG-2022 (764)	RIKEN	X	X
spp.	Las	Lasa, China	MG-0721	RIKEN	X	X
spp.	Lzh	Lanzhou, China	MG-0507	RIKEN	X	X
spp.	Moh	Mohe, China	MG-0670	RIKEN	X	X
spp.	Qiq	Qiqihare, China	MG-0993 (735)	RIKEN	X	X
spp.	Qiq	Qiqihare, China	MG-0992 (734)	RIKEN	X	X
spp.	Tac	Tacheng ,China	MG-0611 (9)	RIKEN	X	X
spp.	Tum	Tumen, China	MG-0980 (722)	RIKEN	X	X
spp.	Wuh	Wuhan, China	MG-0908 (627)	RIKEN	X	X
spp.	Wuh	Wuhan, China	MG-0950 (669)	RIKEN	X	X
spp.	Yaz	Yangzhou, China	MG-0715	RIKEN	X	X
spp.	Zhj	Zhenjiang, China	MG-0713 (80)	RIKEN	X	X
spp.	Zhj	Zhenjiang, China	MG-0714 (81)	RIKEN	X	X
spp.		Amur region, Blagovetsensk, Russia	MG-3064 (182)	RIKEN	X	X
spp.		Birakan Settlement,	MG-3027 (145)	RIKEN	X	X

		Khabarovski region, Western Birobidzan city, Russia				
spp.		Donetsk, Russia	MG-3065 (183)	RIKEN	X	X
spp.		Magadan, Russia	MG-3063 (181)	RIKEN	X	X
spp.		Moscow region, Chevnogolvka, Russia	MG-3056	RIKEN	X	X
spp.		Moscow region, Russia	MG-3058 (176)	RIKEN	X	X
spp.		North Caucasus, Grozny City, Russia	MG-3010 (128)	RIKEN	X	X
spp.		Novii settlement, Russia	MG-3012 (130)	RIKEN	X	X
spp.		Novosibirsk, Russia	MG-3054 (172)	RIKEN	X	X
spp.		Pusan, Korea	MG-0444 (1535)	RIKEN	X	X
spp.		Pusan, Korea	MG-0445 (1536)	RIKEN	X	X
spp.		set.Innokentevka, Russia	MG-3073 (191)	RIKEN	X	X
spp.		Teli settlement, Russia	MG-3004 (122)	RIKEN	X	X
spp.		Vladivostok, Kirova 62, Russia	MG-3034 (152)	RIKEN	X	X
spp.		Vladivostok, Russia	MG-3025 (143)	RIKEN	X	X
spp.		Yu-Sahalinsk, Russia	MG-3047 (165)	RIKEN	X	X

[1]spp., species undetermined

[2]unchecked boxes include mice from the Americas or trapped in duplicate locations or poorly defined sites

Table S2. Primer sequences used for PCR.

Primer	Primer sequence from 5'	Reference sequence (Accession #)	Position in Reference
**MLV env**			
AKVenvF1	TAGAACCTCGCTGGAAGG	AKV MLV (J01998)	5653-5670
AKenvF2	CTGATTCTCGGAGGGGTCAACC	AKV MLV (J01998)	5852-5873
AKenvF1	CACGCCCCCGATAAAACCATC	AKV MLV (J01998)	5762-5782
AKenvR1	GCCAGAGTTAGTGAGACGG	AKV MLV (J01998)	6981-6998
AkvER4	GCCTGGAGTTGTTGGAACTG	AKV MLV (J01998)	7295-7314
AKVspF	CCCCCGATAAAACCATCAT	AKV MLV (J01998)	5766-5784
AKSU7100R	CACAGTAATCGGTGGTGAGG	AKV MLV (J01998)	7087-7106
Emv7480R	CCGTACCAATCCTGTGTG	AKV MLV (J01998)	7489-7504
XE6040F	GAACCTCGCTGGAAAGGAC	AKR40 (MH450109)	5654-5672
XP7400R	GATGGGTTTTGGGAACTGCTCC	AKR6 (MH450110)	6865-6886
Hog3F	CCTGACATTGGGCGGAAG	HoMuLV (KU324805)	1844-1861
Hopol1R	GAAACCAGCCGTTCCCAAG	HoMuLV (KU324805)	3523-3541
HoEnv1F	GGCATTGGGTAAAGGCAGTCC	HoMuLV (KU324805)	5868-5887
HoEnv3R	CAGTCGGTTTAGAATACAGGGT CC	HoMuLV (KU324805)	7654-7677
Xenv150F	CTAATGACAGGACAAACAGC	AKR40 (MH450109)	5932-5951
Xenv400R	CATCCCCATTTGCCTCTGTAG	AKR40 (MH450109)	6165-6185
FrgR1	GCTTCCAGACCTCTCGTTGTAC	Frg3 (AB050721)	53-74
FrgF3	CTCGTGACAGGAGGGTTAGC	*Fv4* (M33884)	160-179
Fv4EF2, ER2	CAACTGCCCTAGTCGCCAC	*Fv4* (M33884)	1595-1614
Fv4-3'R2	CCATCACCCACAAGGTAG	C57BL/6 Chr 12 (NC_000078)	80844616-80844634
Fv4-5'F1	CCTTCTGGGCATTCCGTTAG	C57BL/6 Chr 12 (NC_000078)	80843155-80843174
**Capsid**			
AKp30F2	GGCTTTCCCACTCCGTTTGG	AKV MLV (J01998)	1274-1293
XE1400F	GTCCTCATCACCCACCAG	DG-75 MLV (AF221065)	1395-1412
Emv1840R	CGACCAATGTCTGGAGCG	AKV MLV (J01998)	1838-1855
XMLVp10R	CTCCCCCCTGTCTATCCTGTCTC TG	AKV MLV (J01998)	2088-2112
**Fv1**			
FvF1-F	AGCCGAGTTCTAGGGAAACAA	Fv1b (X97719)	2136-2156
MHRF	TTTAAGGGTGTGGGATAATGG T	Fv1b (X97719)	3003-3024
GT17aR	CATCTATACTATCTTGGTGAG	Fv1b (X97719)	4831-4851
**CAT1**			
CATF1	CTGGTAAGTGGCTGACCCATC	C57BL/6 Chr 5 (NC_000070)	148282801-148282821
CATF2	GCTGTCCTTCTCTTCAGAGC	C57BL/6 Chr 5 (NC_000070)	148279738-148279757

CATR1	CCTATGAACGGTATCACCCAG	C57BL/6 Chr 5 (NC_000070)	148282518-148282538
CATR2	CCTGTGCCAAGAGGAAAAGG	C57BL/6 Chr 5 (NC_000070)	148279471-148279490
CATR3	CCACTGGGCTAAAACACAACG	C57BL/6 Chr 5 (NC_000070)	148278665-148278685
CATR4	CACAGCGCAGGAAGATTCG	C57BL/6 Chr 5 (NC_000070)	148277253-148277271
MCATF	GCGGATCCTAATGGGCTGCAAAAACC	(NM_007513)	280-296
MCATR	CGGGATCCGTCATTTGCACTGGTCC	(NM_007513)	2134-51

Table S3. Accession numbers for MLV XRVs and ERVs and for *Cat1* and *Fv1* gene sequences.

**E-XRVs**	**Accession Number**
AKV	J01998
BM5	AY252102
CasBrE	X57540
CAST17/18	KU324807
FB29	NC_001362
FrMLV	X02794
FrMLV57	LC229035
Graffi-1.2	AB187566
Gross	AY294332
HoMLV	KU324805
MelARV	DQ366148
MoMLV	AF033811
MoMLV-ts1	AF462057
PVC-211	M93134
RaMLV	U94692
RadLV	K03363
SL3-3	AF169256
SRS	AF019230
WN1802B	K01204
**P-XRVs**	
FRMCF	X01679
MoMCF	J02254
RL-MCF-MLV	AF288942
**X-XRVs**	
AKR6	MH450110
AKR40	MH450109
CASTX	KU324803
CWM-S-5X	MH450113
DG-75	AF221065
Du5H	X14576
EKVX	JF908817
Kyushu	MH450114
NZB	EU334447
rv-DBA/2	L37059
SCLC	MH450112
XMRV	DQ399707

**Other XRVs**	
A-MLV	AF411814
CasE#1	KU324804
Cz524	KU324804
**ERVs**	
Cast/EiJ-ERV1	KU324812
Cast/EiJ-ERV3	KU324817
Cast/NcR-ERV	KU324819
CalWM1	KU324810
CalWM2	KU324813
Emv30	KJ668269
ERV1	AF136489
Frg3	ABO50721
Frg1	AB050720
Fv4	M33884
M-RV 1A	AY714500
M-RV 7A	AY714504
M-RV 9B	AY714510
NA1-Mpmv	AY219536
NC2-Pmv	AY219545
NI1-Pmv	AY219557
NQ1-Pmv	AY219567
PreXMRV2	FR871850
Fv1	
CAST/EiJ	FJ603568
Mol/Li	FJ603569
SKIVE	FJ603570
CZECHI/EiJ	FJ603571
LEWES	FJ603572
CalWM	FJ603573
PRAE	FJ603574
***Slc7a1*/CAT1**	
Arvicanthis niloticus	XM_034489669.1
Cavia porcellus	XM_003477412.4
Cricetulus griseus	XM_007652065.4
Fukomys damarensis	XM_010623858.3
Grammomys surdaster	XM_028779808.1
Heterocephalus glaber	XM_004854790.2
Ictidomys tridecemlineatus	XM_040281633.1
Jaculus jaculus	XM_004659984.1
Marmota flaviventris	XM_027928856.1

Marmota marmota	XM_015484182.1
Mastomys coucha	XM_031338779.1
Mesocricetus auratus	NM_001310565.1
Microtus ochrogaster	XM_005344718.3
Mus caroli	XM_021162329.2
M.m.castaneus(Cas/Li)	JN226410
M.m.musculus(CzechI)	JN226407
M.m.molossinus(MOLG)	JN226408
Mus pahari	XM_021186649.2
Mus spicilegus	JN226409.1
Nannospalax galili	XM_029559651.1
NIH3T3	M26687
Peromyscus leucopus	XM_028878053.2
Peromyscus maniculatus	XM_006979618.2
Rattus norvegicus	NM_013111.3
Rattus norvegicusXC	AB066224
Rattus rattus	XM_032886507.1
Octodon degus	XM_004631056.3
Urocitellus parryii	XM_026388381.1

Table S4. Residues under positive selection as identified by separate programs

Gene	Program	Residues under Positive Selection	Reference
Cat1 (All Rodents)	MEME	159V, 226C, 229N, 233V, 237E, 323Q, 365F, 529V, 591T, 598I	Mouse
	FEL	529V, 591T	
	FUBAR	None	
	SLAC	None	
	PAML	233V	
Cat1 (Mice and Hamsters)	MEME	7L, 365F, 598I	Mouse
	FEL	373N	
	FUBAR	373N	
	SLAC	None	
	PAML	599W, 610Q	
E-MLV RBD*env* (without FMR viruses)	MEME	97P, 144A, 169S, 262S	AKV
	FEL	97P, 169S	
	FUBAR	None	
	SLAC	None	
	PAML	92R, 97P, 106G, 108S, 111T, 112P, 203D, 213E, 228Q, 237W	
E-MLV *env* (without FMR viruses)	MEME	21I, 31P, 33T, 35G, 144A, 206T, 293N, 299T, 385L, 400T	AKV
	FEL	10F, 33T, 35G, 293N, 400T	
	FUBAR	27G, 35G	
	SLAC	None	
	PAML	27G, 34L, 237W, 305E, 385L	
X/P MLV *env*	MEME	9P, 31A, 72K, 91D, 94D, 98S, 101G, 107L, 119P, 157G, 161R, 175S, 217T, 228Q, 267T, 294E, 353E, 439K, 476A, 489T, 500A, 502E, 518G, 574G, 575L, 586I, 638P, 639E, 640E	AKR6
	FEL	9P, 91D, 120T, 161R, 175S, 217T, 267T, 294E	
	FUBAR	91D, 161R, 175S, 217T, 267T, 294E	
	SLAC	217T	
	PAML	91D, 175S, 217T	
X/P MLV RBD*env*	MEME	72K, 87D, 88P, 91D, 92I, 107L, 153S, 161R, 217T	AKR6
	FEL	91D, 101G, 217T	
	FUBAR	87D, 91D, 92I, 161R, 175S, 217T	
	SLAC	92I, 217T	
	PAML	84H, 87D, 88P, 91D, 92I, 107L, 161R, 217T	
Capsid	MEME	4L, 10L, 12Y, 35G, 109Q, 110R, 172P, 189D, 243E	AKV
	FEL	4L, 110R	
	FUBAR	4L, 110R	
	SLAC	110R	
	PAML	109Q	
